# Complete Genome Sequence of a Novel Human Gammapapillomavirus Isolated from Skin

**DOI:** 10.1128/genomeA.00833-17

**Published:** 2017-08-24

**Authors:** Rosario N. Brancaccio, Alexis Robitaille, Sankhadeep Dutta, Dana E. Rollison, Nicole Fischer, Adam Grundhoff, Massimo Tommasino, Tarik Gheit

**Affiliations:** a Infections and Cancer Biology Group, International Agency for Research on Cancer, Lyon, France; b Department of Cancer Epidemiology, Moffitt Cancer Center, Tampa, Florida, USA; c German Center for Infection Research, Hamburg, Borstel, Lübeck, Riems, Germany; d Institute for Medical Microbiology, Virology and Hygiene, University Medical Center Hamburg-Eppendorf, Hamburg, Germany; e Heinrich Pette Institute, Leibniz Institute for Experimental Virology, Hamburg, Germany

## Abstract

A novel human papillomavirus (HPV ICB1) was fully characterized from a skin swab by using a sensitive degenerate PCR protocol combined with next-generation sequencing. The L1 open reading frame of HPV ICB1 shares 70.54% nucleotide homology with its closest relative, HPV164, and thus constitutes a novel human gammapapillomavirus.

## GENOME ANNOUNCEMENT

Human papillomaviruses (HPVs) are nonenveloped double-stranded DNA viruses approximately 8 kb in size with an epithelial tropism. HPVs colonize normal skin and mucosa and can induce cutaneous and mucosal lesions (1–4). The L1 gene is well conserved among the papillomaviruses, and thus, it is used for taxonomic classification (5, 6). Here, we report the complete genome sequence of a novel HPV type isolated from a skin swab from a healthy individual.

Degenerate PCR primers (7) were used to screen a cohort of skin samples. The amplicons were purified, pooled, and sequenced by next-generation sequencing (NGS) using the NEBNext Ultra DNA library prep kit and MiSeq reagent kit version 2 (Illumina). NGS analysis revealed the presence of a sequence of approximately 205 bp from a putative new HPV.

The complete viral genome of a new HPV type (HPV ICB1, 7,233 bp), with a G+C content of 38.09%, was obtained by DNA amplification using multiply primed rolling circle amplification (RCA) according to the manufacturer’s instructions (illustra TempliPhi 100 amplification kit; GE Healthcare, USA). RCA was combined with long-range PCR (LA Taq polymerase; TaKaRa Bio, Japan) performed with outward-directed primers specific for the putative new HPV (forward primer, 5′-CATTTTGCTCATCATCACATGGCC-3′; reverse primer, 5′-CTGGTGACTGTCCTCCTATCC-3′). An amplicon of approximately 8 kb in size was cloned in the pCR-XL-TOPO vector using the TOPO-XL PCR cloning kit (Invitrogen, USA) and sequenced by a primer walking strategy (GATC Biotech, Germany). The sequence was validated using a proofreading polymerase, followed by Sanger sequencing.

HPV L1 sequences that share less than 90% sequence similarity to the closest papillomavirus type are traditionally considered to be distinct HPV types (5, 8). The L1 open reading frame (ORF) of HPV ICB1 showed 70.54% nucleotide homology (9) with its closest HPV type, HPV164, belonging to species gamma-8 (GenBank accession no. JX413106). In addition, according to a BLASTn search, the overall nucleotide homology between HPV ICB1 and HPV119 (gamma-8; GenBank accession no. GQ845441) was 69%. Analysis of the HPV ICB1 genome showed the presence of five early (E1, E2, E4, E6, and E7) and two late (L1 and L2) ORFs. The E5 ORF was absent. The long control region between L1 and E6 has a length of 514 bp and contains the TATA box (TATAAA), one polyadenylation site (AATAAA) for L1 and L2 transcripts, and four consensus palindromic E2-binding sites (ACC-N_6_-GGT). Like all HPV types, E6 and E7 have zinc-binding domains [CxxC(x)29CxxC] containing two and one zinc-binding domains, respectively. In addition, E7 contains an LxSxE retinoblastoma (RB)-binding motif (10). Analysis of the E1 ORF revealed the presence of a putative ATP-binding site of the ATP-dependent helicase, a GPPDTGKS motif (11). Moreover, two cyclin interaction RXL motifs (10, 11) have been localized in the E1 protein. In conclusion, analysis of the complete nucleotide sequence showed that HPV ICB1 shares the features of other known gammapapillomaviruses.

### Accession number(s).

The complete genome sequence of HPV ICB1 is available in GenBank under the accession number MF356498.
